# Sonic hedgehog promotes chondrogenesis of rabbit bone marrow stem cells in a rotary cell culture system

**DOI:** 10.1186/s12861-019-0198-4

**Published:** 2019-08-12

**Authors:** Liyang Chen, Gejun Liu, Wenjun Li, Xing Wu

**Affiliations:** 1 Department of Orthopaedics, Tenth People’s Hospital of Tongji University, Tongji University, Shanghai, 200072 China; 2 School of Medicine, Tongji University, Shanghai, 200072 China

**Keywords:** Shh, Bone marrow stromal cells, Rotary cell culture system, Chondrogenesis, Tissue engineering

## Abstract

**Background:**

Sonic hedgehog (Shh) is an important signalling protein involved in the induction of early cartilaginous differentiation. Herein, we demonstrate that Shh markedly induces chondrogenesis of rabbit bone marrow stromal cells (BMSCs) under microgravity conditions, and promotes cartilage regeneration.

**Results:**

In the rotary cell culture system (RCCS), chondrogenic differentiation was revealed by stronger Toluidine Blue and collagen II immunohistochemical staining in the Shh transfection group, and chondroinductive activity of Shh was equivalent to that of TGF-β. Western blotting and qRT-PCR analysis results verified the stronger expression of Sox9, aggrecan (ACAN), and collagen II in rabbit BMSCs treated with Shh or TGF-β in a microgravity environment. Low levels of chondrogenic hypertrophy, osteogenesis, and adipogenesis-related factors were detected in all groups. After transplantation in vivo, histological analysis revealed a significant improvement in cartilage and subchondral repair in the Shh transfection group.

**Conclusions:**

These results suggested that Shh signalling promoted chondrogenesis in rabbit BMSCs under microgravity conditions equivalent to TGF-β, and improved the early stages of the repair of cartilage and subchondral defects. Furthermore, RCCS provided a dynamic culture microenvironment conducive for cell proliferation, aggregation and differentiation.

## Background

Articular cartilage coverage on the surface of joints minimises friction and helps to withstand repetitive loads. Chondrocytes and extracellular matrix (ECM) are the main components of hyaline-type cartilage [1]. ECM is composed of collagen II, aggrecan (ACAN), chondroitin sulfate, and other glycosaminoglycans that are important for maintaining the biomechanical properties of articular cartilage [2, 3]. As physical activity is increasing among people of all ages, articular cartilage injuries are also increasing. Self-healing of cartilage damage is difficult because of a lack of vascular, nervous, and lymphatic systems in articular cartilage [4], and osteoarthritis (OA) often follows primary injuries [5].

Most previous studies related to cartilage regeneration have focused on cell-based therapy using scaffolds or growth factors in a rabbit osteochondral defect model [6, 7]. Unfortunately, autologous chondrocyte implantation is not suitable for treating cartilage defects due to inconvenient sampling, limited sources and poor proliferation ability in vitro [7]. BMSCs have attracted much attention in regenerative medicine and tissue engineering due to their multilineage differentiation, particularly in chondrogenic differentiation [1, 8, 9]. However, the mechanisms regulating their chondrogenic potential are not clearly understood.

Studies to identify the growth factors or molecules that regulate the chondrogenic potential of BMSCs are crucial for optimising their therapeutic use in cartilage disorders. Sonic hedgehog (Shh), a member of the hedgehog protein family, plays a central role in a variety of developmental events and influences patterning of the eye, wing, and leg in Drosophila embryos [10]. Studies have shown that Shh plays an important role in inducing early chondrogenic differentiation during normal chondrogenesis in vivo [10, 11]. Shh has a positive effect on cartilage repair, causing increased expression of Patched (Ptc), Gli1, and Sox9 in Shh-transfected chondrocytes after 48 h [11].

The specific growth environment is an important factor in chondrogenic differentiation. Conventional approaches for chondrogenic differentiation of MSCs are typically limited to two-dimensional or pellet cell culture environments [12]. However, these traditional culture methods cannot effectively maintain the induced phenotype of stem cells. Studies have shown that the three-dimensional (3D) culture environment and appropriate mechanical stimulation are both important for chondrogenic differentiation of MSCs [13]. The rotary cell culture system (RCCS) as a new 3D microgravity culture system have used for culture and induce chondrogenic differentiation [12, 14]. This method can establish a suspension orbit in the RCCS container, while simultaneously balancing gravity, buoyancy, and shear force, generating a microgravity environment conducive for cell aggregation [12]. Appropriate mechanical stress helps to maintain cellular phenotype and function. Using this technique, adipose-derived stem cells can rapidly attach, extend and proliferate [13].

Therefore, in this work, we amplified and induced chondrogenic differentiation of BMSCs transfected with Shh using a combined bioreactor and microcarrier approach. The main aim was to explore the effect of Shh on the chondrogenesis of BMSCs under microgravity conditions, and assess the regeneration of cartilage.

## Results

Passage 2 rabbit BMSCs transfected with Shh adenovirus or green fluorescent protein (GFP) adenovirus plasmid were incubated in the RCCS environment. Bioreactor culturing of adherent cells requires a growth surface that allows cells to remain suspended in the culture medium. Cytodex 3 microcarriers were used to provide a stable but non-rigid surface to which BMSCs can easily attach from stirred cultures. GFP-transfected BMSCs cultured with 10 ng/mL TGF-β3 served as a positive control. Gene expression and histological staining analyses were performed to assess the effects of Shh and TGF-β on chondrogenesis in the RCCS environment. Undifferentiated BMSCs transfected with Shh adenovirus plasmids were implanted in rabbit osteochondral defects after 3 days of expansion in RCCS. Histological analyses were carried out to analyse cartilage repair at 6 and 12 weeks after surgery.

### Morphology of BMSCs on the Cytodex 3 microcarrier surface

**Fig. 1:**
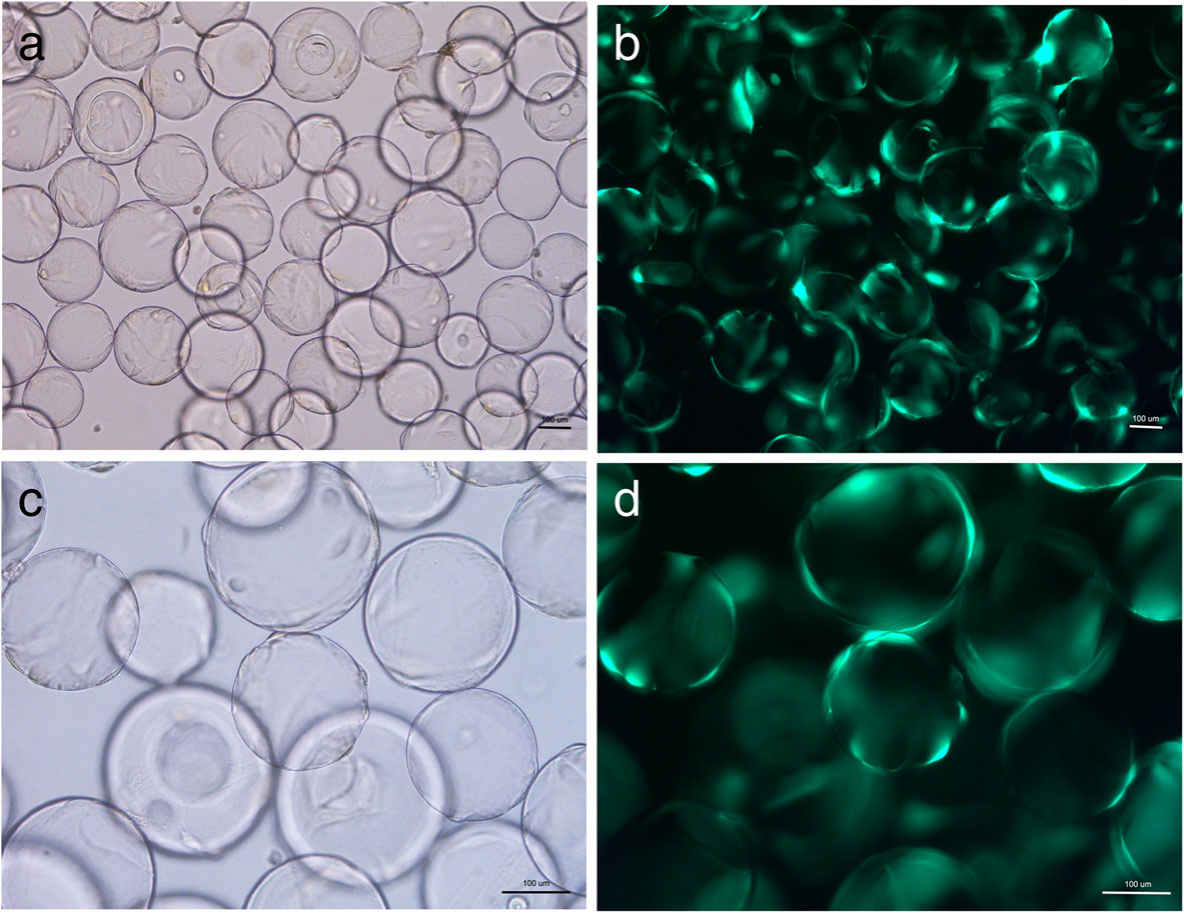
BMSCs after transfected with Shh viral plasmids adhere to the the surface of Cytodex 3 microcarriers observed via inverted microscope (**a** and **c**) and fluorescence microscopy (**b** and **d**). Scale bar: 100 μm

After 24 h, > 80% of rabbit BMSCs had attached to the microcarriers after incubation in a 10 mL RCCS cell culture container, and < 2% of dead cells appeared in the culture medium. Adherent cells were visible on a different focal plane, and cells were irregular or spindle-shaped when viewed under an inverted microscope (Fig. 1). Green fluorescence was observed after cells were transfected with adenovirus plasmid. Cell-microcarrier complexes appeared as lantern-like objects via fluorescence microscopy (Fig. 1b, d).

### Expression of Shh and hedgehog signalling molecules following transfection

**Fig. 2:**
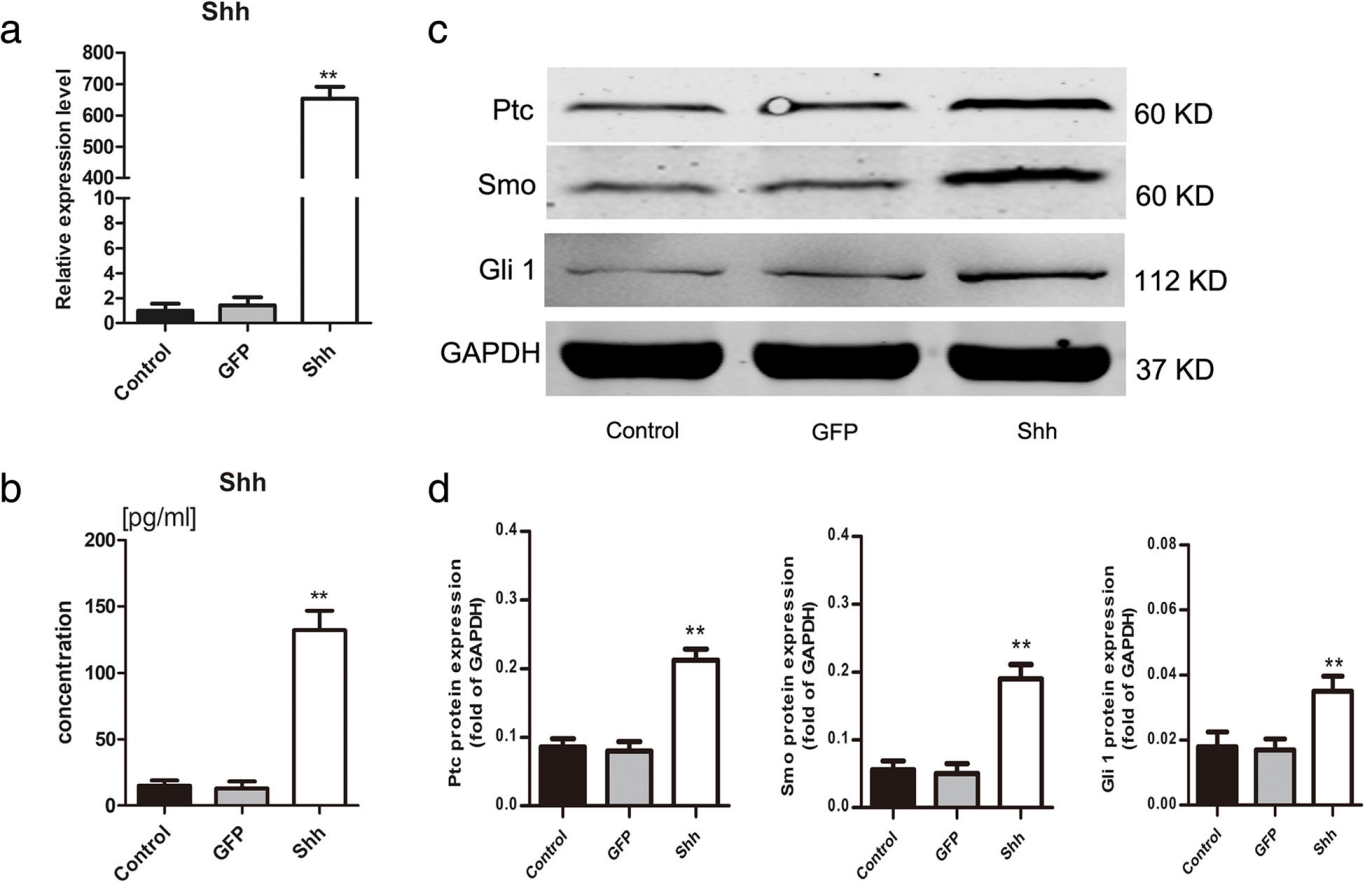
The effect of Shh on hedgehog signaling. **a**, **b** qRT-PCR and ELISA were used to demonstrate Shh expression levels after transfected with Shh viral plasmids. **c** Expression levels of Ptc, Smo and Gli1 were detected by western blotting. **d** Results were normalized to GAPDH protein expression. The representative results were from three independent experiments. Significant differences between the control group are indicated by * *p* < 0.05, **p < 0.01

ELISA and qRT-PCR experiments clearly revealed high expression levels of Shh in Shh-transfected BMSCs but not in GFP-transfected and non-transfected BMSCs (*p* < 0.001; Fig. 2a, b). The Shh signalling pathway was also analysed by western blotting after transfection. The results showed that levels of Ptc, smoothened (Smo), and Gli1 were higher in Shh-transfected BMSCs after 72 h (*p* < 0.01; Fig. 2c, d).

### Cell viability and proliferation on Cytodex 3

**Fig. 3:**
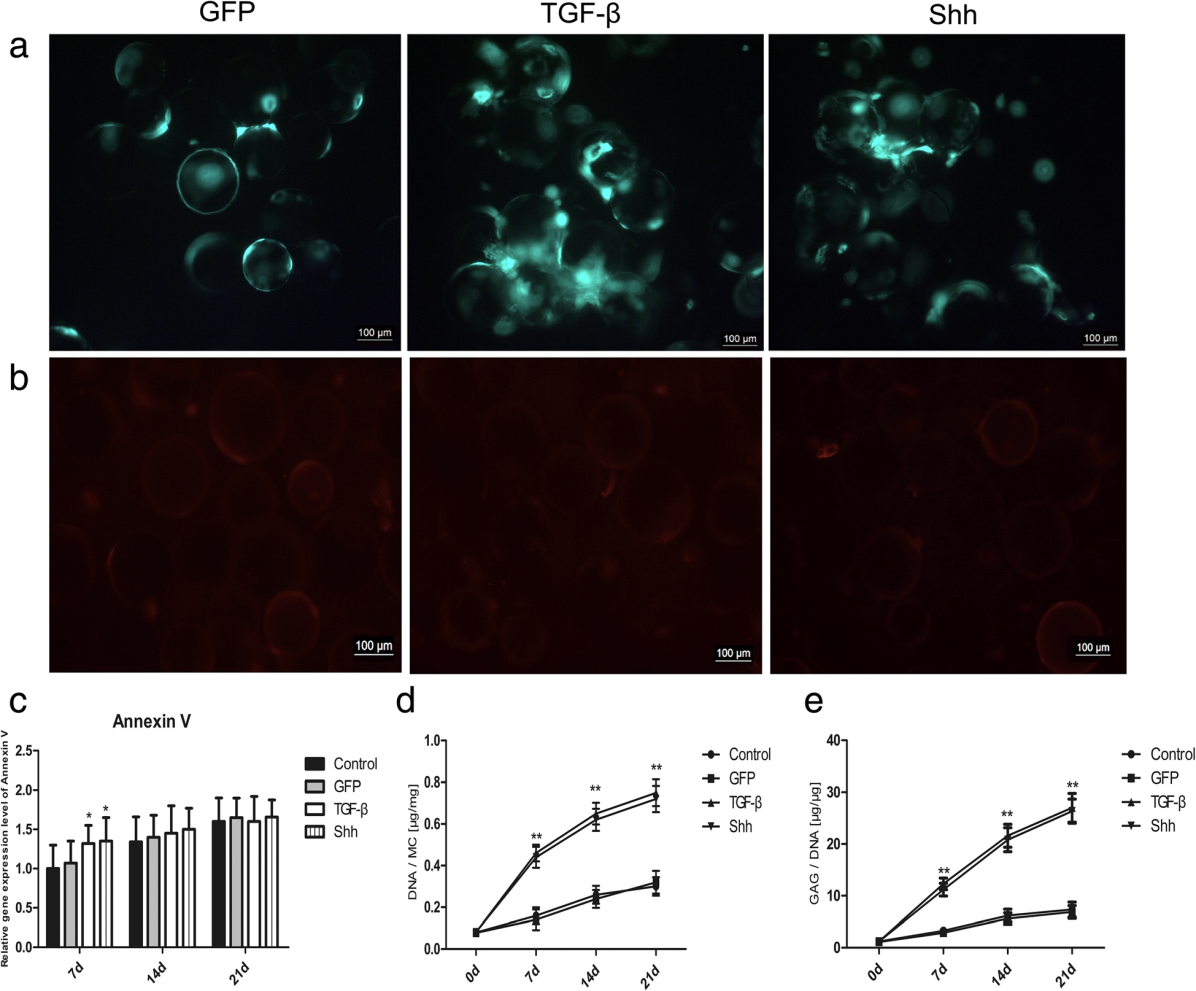
BMSCs viability and proliferation on cytodex 3. **a** BMSCs transfected with Shh or GFP viral plasmids revealed green fluorescence after 21 days of culture. GFP-transfected BMSCs treated with TGF-β served as a positive control. **b** Annexin V-PE immunofluorescence staining of BMSCs on cytodex 3 at day 21. Dead cells are labeled in red. **c** RT-PCR analysis of Annexin V on days 7,14 and 21 during differentiation induction. **d**, **e** Comparison of DNA and GAG/DNA ratio at different time points. MC, microcarrier. Significant differences between the control group are indicated by * *p* < 0.05, **p < 0.01

Single fluorescence staining with annexin V-PE was used to assess cell viability and apoptosis following chondrogenesis. After induction for 21 days, BMSCs treated with Shh or TGF-β displayed equivalent high levels of green fluorescence (Fig. 3a). By contrast, annexin V-positive (red fluorescence) cells were scarce in all groups (Fig. 3b). Meanwhile, mRNA expression levels of annexin V gradually increased in all groups, but not significantly during chondrogenic differentiation (Fig. 3c).

The DNA and sGAG content in BMSCs treated with Shh or TGF-β were significantly upregulated in vitro over time (Fig. 3d, e), indicating continuous cell proliferation and sGAG accumulation.

### Histological assessment and immunohistochemical analyses of chondrogenesis

**Fig. 4:**
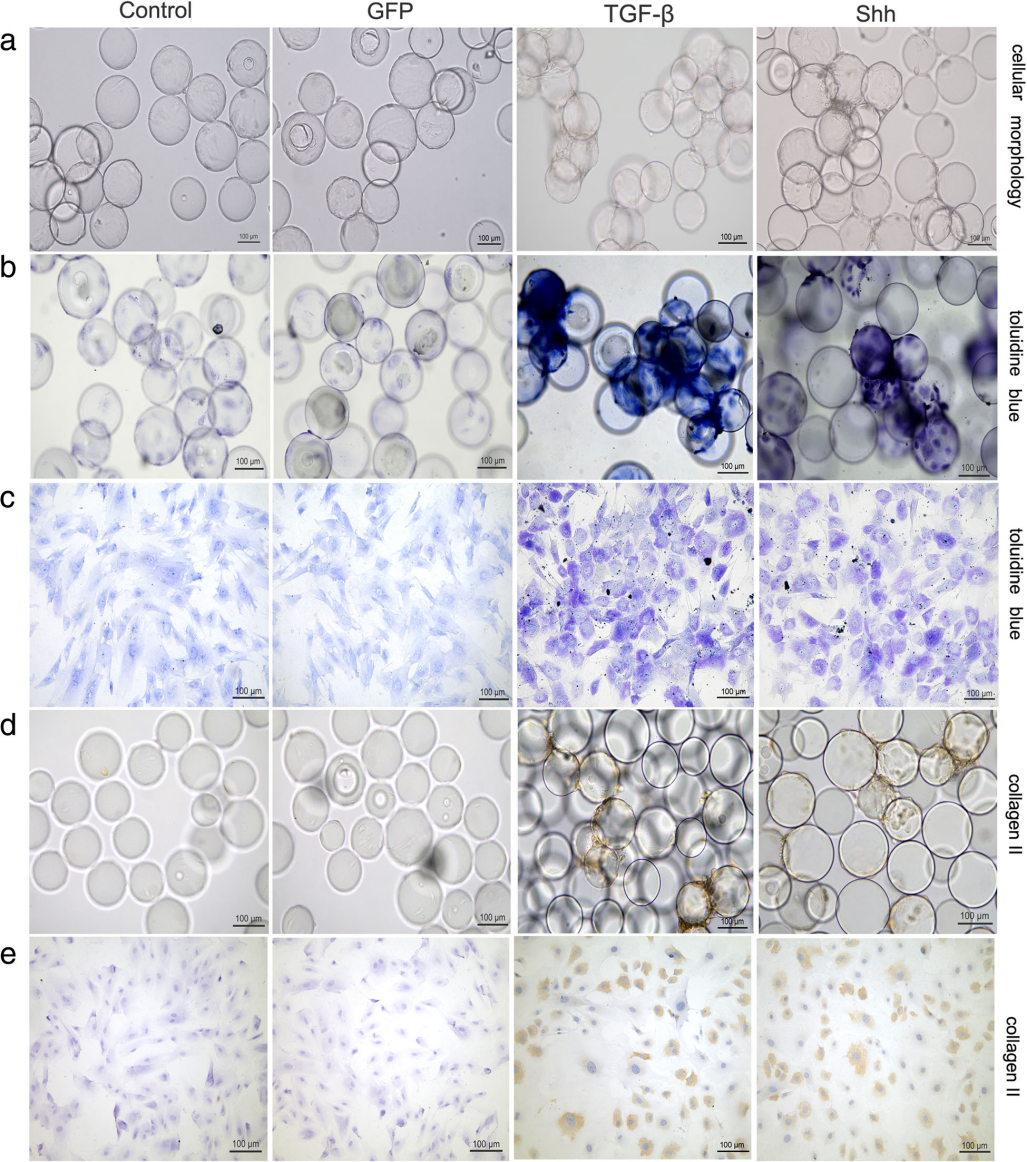
The effect of Shh on chondrogenesis after 21 days of differentiation induction in the RCCS environment. **a** Cellular morphology. **b** Toluidine blue staining on cell-microcarrier complexes. **c** Toluidine blue staining on the cells after inoculated onto slides. **d** Collagen II immunohistochemical staining on cell-microcarrier complexes. **e** Collagen II immune-histochemical staining on the cells after inoculated onto slides. Scale bar: 100 μm

In the RCCS environment, a large number of cells covered the surface of the microcarrier in both Shh and TGF-β treatment groups, and microcarriers overlapped with each other, creating a rough and uneven surface (Fig. 4a). Numerous cellular connections were observed between microcarriers, and only a few dead cells were present in the medium. Chondrogenic differentiation was observed as strong metachromatic staining for proteoglycans with Toluidine Blue in BMSCs treated with Shh or TGF-β, compared with non-transfected and GFP-transfected BMSCs (Fig. 4b, c). After induction, the secretion of ECM was increased significantly, and it was difficult to observe single cells. Large connectomes were observed between the microcarriers, and the microcarriers grew in clusters.

Correspondingly, immunohistochemistry revealed high expression of the cartilage matrix protein collagen II on day 21 of culturing in BMSCs treated with Shh or TGF-β, compared with non-transfected and GFP-transfected BMSCs (Fig. 4d, e). There were no clear differences between the Shh and TGF-β groups in terms of chondrogenic appearance.

### Effect of Shh on expression of cartilage-specific markers in the RCCS

**Fig. 5:**
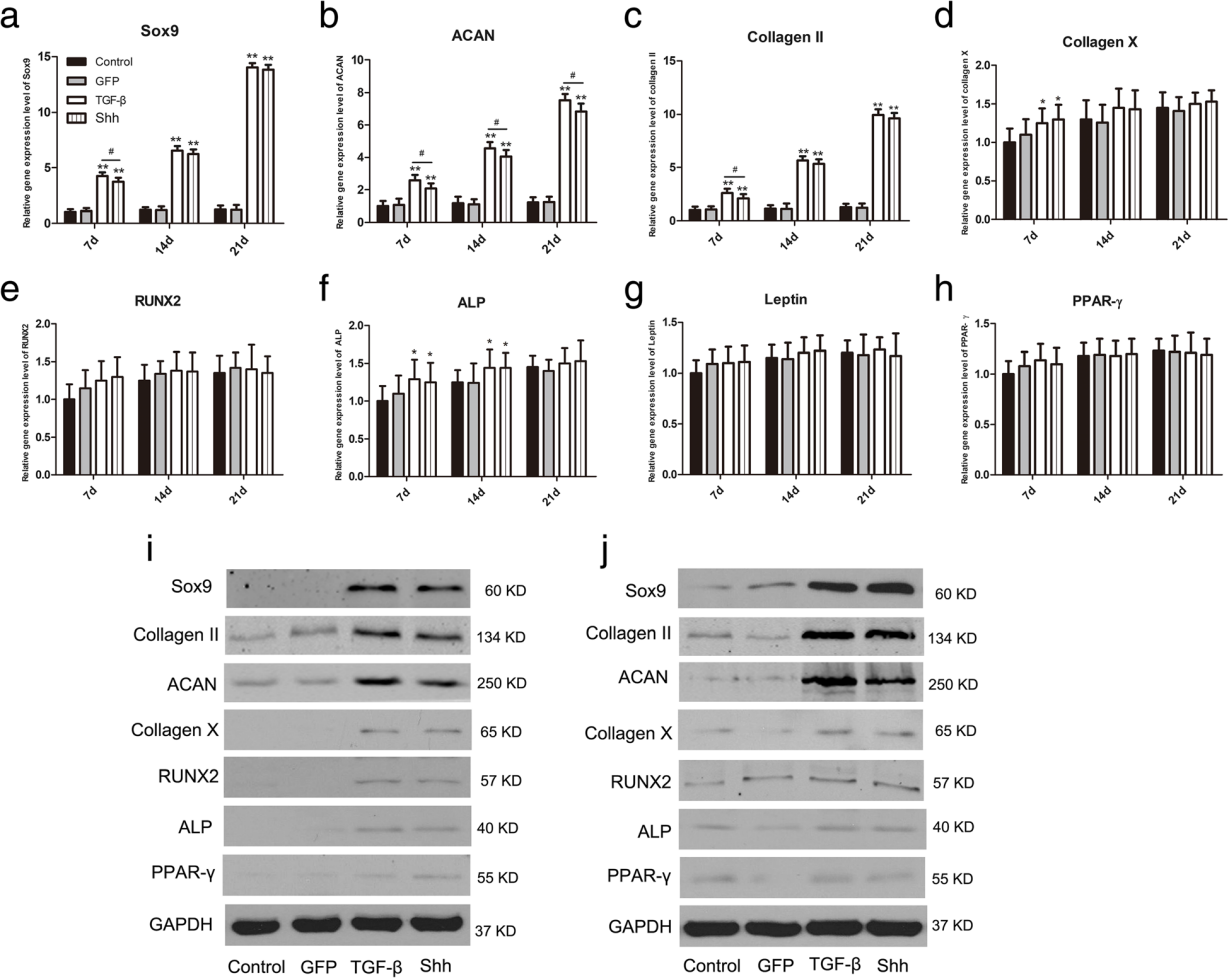
The effect of Shh on cartilage related markers during differentiation induction in the RCCS environment. **a**-**h** RT-PCR analysis of Sox9, ACAN, collagen II, collagen X, RUNX2, ALP, PPAR-γ and leptin on days 7,14 and 21 during differentiation induction. **i**, **j** Expression of Sox 9, collagen II, ACAN, collagen X, RUNX2, ALP and PPAR-γ in the RCCS dimensional environment on days 10 and 21 of chondrogenesis. The representative results were from three independent experiments. Significant differences between the control group are indicated by * *p* < 0.05, **p < 0.01; differences between Shh and TGF-β transfection groups are indicated by ^#^ *p* < 0.05 or ^##^ *p* < 0.01

Expression levels of cartilage-specific marker genes were investigated using qRT-PCR and western blotting, and the results are shown in Fig. 4. In the RCCS environment, Sox9, ACAN and collagen II mRNA levels in Shh and TGF-β groups were significantly upregulated compared to GFP-transfected and non-transfected BMSCs at days 14 and 21 of culturing, in which chondrogenic genes were expressed at low levels (*p* < 0.05; Fig. 5a–c). There were no clear differences between Shh and TGF-β groups in terms of expression levels of cartilage-specific marker genes.

Similarly, expression levels of chondrogenic hypertrophy-, osteogenesis- and adipogenesis-related markers containing collagen X, RUNX2, ALP, PPAR-γ and leptin mRNAs were slightly elevated in all groups on days 7, 14 and 21 of culturing (Fig. 5d–h). Again, major differences between Shh and TGF-β groups could not be detected.

Protein levels of chondrogenic-related markers were measured by western blotting analysis after 10 and 21 days of culturing. Expression of Sox9, ACAN and collagen II was significantly increased in Shh and TGF-β groups at day 21 of differentiation (Fig. 5i, j), while levels of collagen X, RUNX2, ALP and PPAR-γ were lower in all groups (Fig. 5i, j).

### In vivo transplantation of Shh-transfected cells

**Fig. 6:**
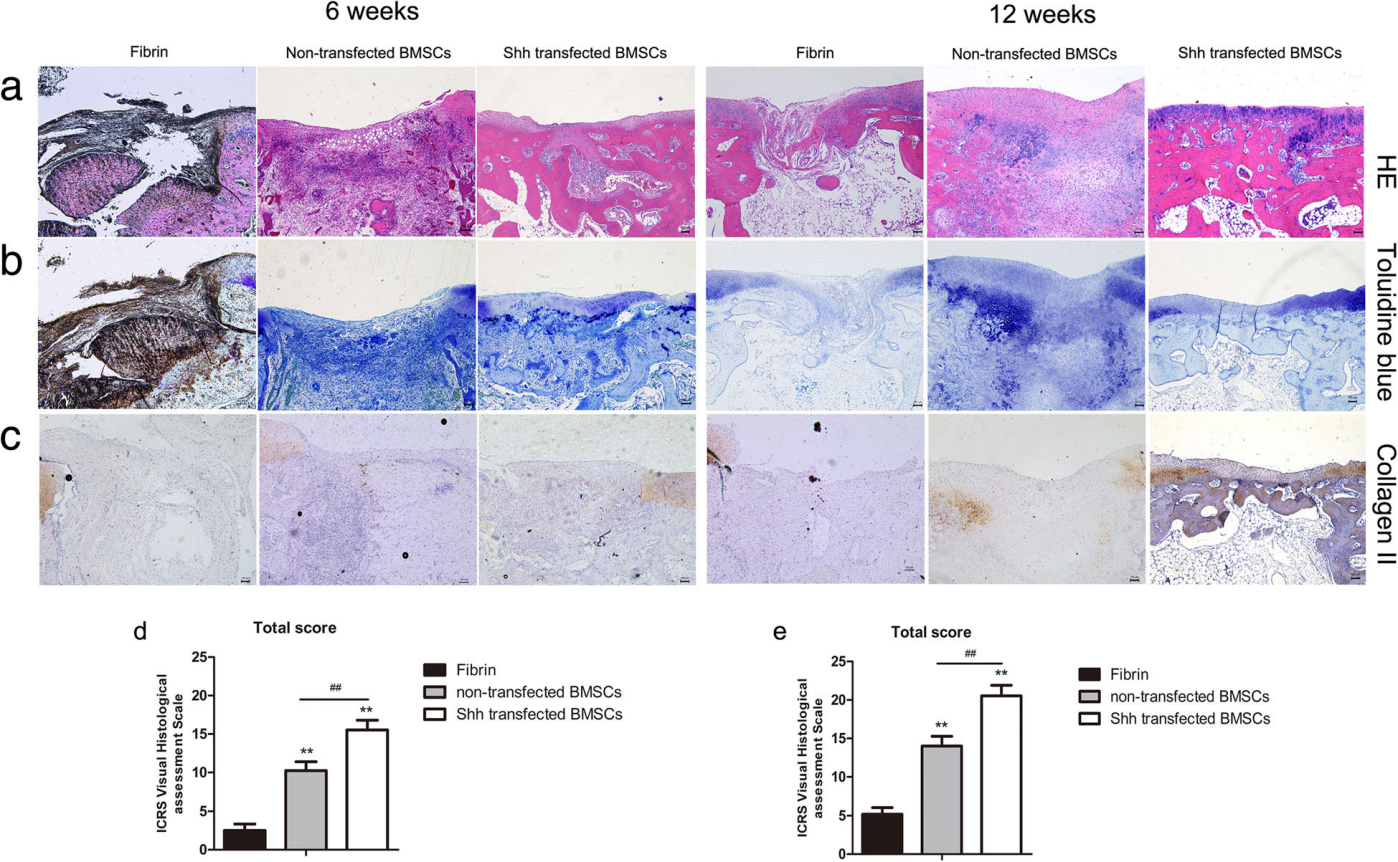
The effect of Shh on cartilage repair. **a** Hematoxylin & eosin staining of at 6 and 12 weeks after surgery. **b** Toluidine blue staining. **c** Immunostaining for collagen II. **d** ICRS Visual Histological Assessment Scale for repaired cartilage at 6 weeks. **e** ICRS Visual Histological Assessment Scale for repaired cartilage at 12 weeks. Significant differences between the fibrin group are indicated by * *p* < 0.05, **p < 0.01. Significant differences between the fibrin group are indicated by *p < 0.05 or ** *p* < 0.01; differences between Shh-transfected BMSC group and non-transfected BMSC group are indicated by ^#^ *p* < 0.05 or ^##^ *p* < 0.01. Scale bar: 100 μm

Undifferentiated Shh-transfected BMSCs were transplanted into an osteochondral defect model after 3 days of expansion in RCCS. At 6 weeks after surgery, cartilage defects were partially repaired in the non-transfected BMSC group, and displayed an unsmooth interface and distinct borders, as shown by HE staining (Fig. 6a). In the fibrin group, no staining was detected. By contrast, defects in the Shh-transfected BMSC group were well integrated into the peripheral tissues, except for a slight central depression, which was superior to the non-transfected BMSCs group at the same time point. Joint surfaces of defects in the Shh-transfected BMSCs group were filled with numerous round or spindle-shaped cells, and a small amount of cartilage-like ECM, as shown by HE and Toluidine Blue staining (Fig. 6a, b), while non-transfected BMSCs contained many spindle-shaped fibroblasts. Histological scoring was higher for the Shh-transfected BMSCs group than the non-transfected BMSCs group (*p* < 0.05; Fig. 6d).

At 12 weeks after surgery, defects in the Shh-transfected BMSCs group were better repaired than those in the non-transfected BMSCs group (Fig. 6a), with a smoother surface and more intimate connections with the surrounding normal tissue. Furthermore, subchondral bone was regularly filled the scaffold and was better connected to surrounding bone in the Shh-transfected BMSCs samples. Neotissue in the non-transfected BMSCs group displayed much less intense staining with Toluidine Blue than the Shh-transfected BMSCs group, which was similar to the surrounding normal cartilage, indicating better cartilage repair (Fig. 6b). Again, histological scoring was higher for the Shh-transfected BMSCs group than the non-transfected BMSCs and fibrin groups (Fig. 6f).

The results of immunohistochemical staining of sections with collagen II antibody are shown in Fig. 6c. In samples from the Shh-transfected BMSCs group, regenerated tissue stained positive for collagen II, while repaired tissue in the non-transfected BMSCs group was negative for collagen II at 12 weeks.

## Discussion

Due to the limited ability to self-regenerative, repair of cartilage is challenging [15, 16]. The present study showed that Shh regulated chondrogenesis in BMSCs under microgravity conditions, and significantly improved the early stages of cartilage repair. In the RCCS environment, Shh was comparable to TGF-β in its ability to significantly promote the chondrogenic potential of BMSCs by stimulating proliferation and inducing the expression of chondrogenic markers.

Shh is a member of the hedgehog protein family that also includes Indian hedgehog and Desert hedgehog [17]. Hedgehog signalling occurs through interactions with cell surface receptors Ptc and Smo, and subsequently via Gli transcription factors that modify the expression of downstream target genes [18]. In this work, expression of hedgehog signalling molecules Ptc, Smo, and Gli1 was upregulated in the Shh-transfected BMSCs compared with other groups.

Some studies have shown that microgravity culturing can promote the proliferation and differentiation of MSCs [13, 19, 20]. Cytodex 3 microcarriers have a larger specific surface than typical culture flask, and can support a cell density and seed cell number that is hundreds of times greater, making them particularly suitable for preparing a large volume of tissue [13, 20]. A thin layer of collagen on the surface of Cytodex 3 allows cells to attach easily from stirred cultures. Under the RCCS environment, gravity, buoyancy, and shear force can reach a balance during rotation to create a microgravity environment that is conducive for cell aggregation. During microcarrier culturing, collision between microcarriers can increase interactions between cells and promote the aggregation of cells. It has been reported previously that cell-cell and/or cell-matrix interactions may regulate the differentiation and metabolic state of chondrocytes [21]. In the present study, BMSCs treated with Shh or TGF-β showed positive Toluidine Blue staining, collagen II immunohistochemical staining, and upregulation of cartilage-specific markers after 21 days of induction in the RCCS environment. These results are consistent with previous observations [13, 19]. Yin et al. reported that BMSCs can proliferate rapidly on the surface of cartilage ECM-derived particles with high viability [19].

A previous study indicated that nutrients can be effectively distributed in the RCCS environment, and the exchange of nutrients and metabolites is also significantly improved throughout the microenvironment [22]. Therefore, the RCCS environment can maintain a stable inducing environment and effectively promote the proliferation and chondrogenesis of BMSCs. In our investigation, less than 2% of dead cells were found in the medium under microgravity culture. In our previous study, we demonstrated that Shh facilitates chondrogenic differentiation of BMSCs during the early stages in a traditional 2D culture environment, and also promotes hypertrophic differentiation and aging [23]. In this work, levels of cartilage hypertrophy- and osteogenesis-related genes were also lower in Shh and TGF-β BMSCs groups. This phenomenon may be related to stress generated by the flow of the culture medium, and may also be related to collision between microcarriers that stimulates cell aggregation and increases interactions between cells.

GFP-transfected and non-transfected BMSCs cultured in the RCCS environment without added exogenous growth factors showed minimal Toluidine Blue and collagen II immunohistochemical staining. Delivery of Shh via adenoviral vectors led strong chondrogenesis in rabbit BMSCs under microgravity conditions, and Shh was equal in potency to TGF-β. This suggests that Shh can effectively induce chondrogenic differentiation of BMSCs after 21 days of induction without exogenous growth factors. Our results are consistent with those of a previous study using recombinant hedgehog proteins to stimulate cartilage production in vitro [10]. In this previous study, expression of cartilage-related markers was slightly increased after treatment with r-Shh in a 2D environment. However, in our present study, expression of ACAN and collagen II was significantly higher in Shh-transfected cells on day 21 in the RCCS, suggesting that Shh can effectively promote chondrogenic differentiation of BMSCs in a microgravity environment.

Both bone and cartilage originate from MSCs. However, it is difficult to regulate the directional differentiation of artificial transplanted MSCs into osteoblasts or chondrocytes [24]. To assess the effect of Shh on cartilage repair, undifferentiated Shh-transfected BMSCs were transplanted into an osteochondral defect model after 3 days of expansion in RCCS. Because a layer of denatured collagen covering the surface of microcarriers is easily digested by a variety of proteases, including trypsin and collagenase, cells can be easily digested from microcarriers, and maintaining maximum cell viability and integrity can be challenging [13]. Previous work revealed that harvesting MSCs from Cytodex 3 does not affect cell activity or proliferation [25, 26]. Implantation of non-transfected BMSCs appeared to promote recovery in the defected region. Furthermore, Shh-transfected BMSCs showed a higher recovery capacity, with the scaffold regularly filled with subchondral bone, and good integration with surrounding normal bone tissue at 12 weeks, unlike non-transfected BMSCs. This indicated that Shh-transfected BMSCs may participate in further regeneration of subchondral defects. Better surface integrity and increased proteoglycan content were observed in the regenerated tissue, verified by histological analysis of the Shh-transfected BMSCs group.

The application of appropriate cell scaffolds combined with allogenic MSCs and bioactive molecules in the treatment of subchondral defects have been investigated in many studies. In the present study, we observed rapid subchondral regeneration in the Shh-transfected BMSCs group at week 6 after implantation. Achieving adequate connections between repaired cartilage and the surrounding normal cartilage during the repair process is challenging, particularly for deep subchondral bone formation [5]. Comparison of groups with BMSCs or scaffolds alone revealed that scaffolds containing BMSCs transfected with Shh showed better integrated cartilage regeneration and subchondral bone formation. A study conducted by Lin et al. demonstrated the curative effect of delivering Shh-transfected dedifferentiated chondrocytes with fibrin glue in osteochondral defect models [11]. Although MSCs have long been used in tissue engineering, little is known about the fate of implanted cells in vivo, and how they affect new tissue repair. These limitations should be resolved in further investigations.

## Conclusions

In conclusion, our results demonstrated that Shh regulated BMSC chondrogenesis under microgravity conditions equivalent to TGF-β, and significantly improved the early stages of the repair of cartilage and subchondral defects. Furthermore, the RCCS provided a dynamic culture microenvironment that is conducive for cell proliferation, aggregation and differentiation.

## Methods

### Generation and propagation of recombinant adenoviral vectors

pDC316-mCMV-ZsGreen1 construct carrying the rabbit Shh target gene (Gene ID: 100352774) was used as a vector, and HEK293 cells were used for viral packaging. The concentration of adenovirus was 10^11^ plaque-forming units (pfu)/mL. An adenovirus plasmid containing enhanced GFP was purchased from Genomeditech (Shanghai, China), and the concentration was 10^11^ pfu/mL.

### Transfection and culturing of rabbit BMSCs

BMSCs were isolated from 4-week-old female New Zealand White rabbits (Shanghai Jambo Biological Technology Co., Ltd., Shanghai, China). All experimental procedures were approved by the Care of Experimental Animals Committee of Tenth People’s Hospital of Tongji University. Rabbits were sedated by intra-muscular injection of ketamine (10 mg/kg) and xylazine (3 mg/kg), and were then sacrificed with pentobarbital (120 mg/kg). After sacrifice, bone marrow aspirates were taken from the iliac crest and femur bones. Briefly, cells were collected, centrifuged, resuspended, and cultured in fresh complete medium consisting of L-DMEM (Gibco, Grand Island, NY, USA) containing 10% FBS (Gibco) and 1% penicillin/streptomycin. BMSCs were used for subsequent detection at passage 2 [12].

Transfection methods were based on our previous study [12, 23]. Shh adenovirus or GFP adenovirus plasmids were transfected into rabbit BMSCs at 50 pfu/cell, and the medium was replaced after 4 h of transfection.

### Measurement of Shh expression

qRT-PCR was used to measure Shh mRNA expression using three randomly selected samples from each group. Shh concentrations in the cell culture medium from each group were detected by ELISA with a rabbit SHH protein ELISA kit (JianglaiBio, Shanghai, China) as described previously [18].

### Chondrogenic differentiation in a RCCS

Cytodex 3 microcarriers (GE Healthcare Life Sciences, Little Chalfont, UK) with a thin layer of collagen were used to provide a stable but non-rigid surface that allows adhesion of BMSCs. Disinfection and sterilisation of microcarriers was based on a previous study [13]. After sterilisation by autoclaving at 121 °C for 20 min, microcarriers were stored at 4 °C.

For chondrogenic differentiation, the differentiation medium included H-DMEM (Hyclone, Pittsburgh, USA), 1% ITS (Gibco), 1% penicillin-streptomycin (Gibco), 100 μg/mL sodium pyruvate, 10^− 7^ M dexamethasone, 50 μg/mL ascorbate, and 40 μg/mL L-proline (Sigma-Aldrich, St. Louis, USA). GFP-transfected BMSCs cultured with 10 ng/mL TGF-β3 (PeproTech EC Ltd., London, UK) served as a positive control. BMSCs were digested by trypsin after 24 h of transfection. Culturing in the RCCS was based on our previous study [23]. Cells at a density of 4 × 10^5^ cells/mL, microcarriers at 5 mg/mL, and chondrogenic differentiation medium were mixed thoroughly and inoculated into the RCCS container. The RCCS container was initially set at a speed of 10–12 rpm to ensure thorough cells contact with the microcarriers. The rotation speed was adjusted to 12–14 rpm after 24 h, cell-microcarriers cannot contact with the container wall, and instead maintained in free-fall during rotation. Culture medium was changed every 2 days during the 21 days of induction.

To harvest cells, a sample of cell-microcarrier constructs was extracted from the container using a syringe, washed with phosphate-buffered saline (PBS) two or three times, and cells were removed from microcarriers by digestion with 0.25% trypsin-EDTA. Harvested cells were used for the subsequent experiment.

### Evaluation of DNA and GAG content

All BMSC-microcarrier constructs were washed with PBS and stored at − 80 °C immediately. For DNA and GAG quantification, BMSC-microcarrier constructs were digested with 0.125 mg/mL papain at 65 °C overnight. DNA content was assessed by a Quant-iT™ Picogreen® dsDNA Assay kit (Life Technologies, Carlsbad, USA), and GAG production was determined using a Blyscan Sulfated Glycosaminoglycan Assay kit (Biocolor Ltd., UK) accordance to the manufacturer’s instructions.

### RNA isolation and reverse transcription analysis

Total RNA was extracted from cells using TRIzol (Invitrogen, Carlsbad, CA, USA) on days 7, 14 and 21. RNA was quantified using a NanoDrop spectrophotometer (Thermo, USA). 1 μg of total RNA from each sample was used for reverse transcription using a PrimeScript RT-PCR kit (TaKaRa, Shiga, Japan). qRT-PCR was performed with an initial denaturation at 95 °C for 3 min, followed by 40 cycles at 95 °C for 3 s and 60 °C for 30 s. β-2-Microglobulin (*B2M*) was selected as an internal control [27, 28]. Relative mRNA expression of target genes (collagen II, ANCN, Sox9, collagen X, RUNX2, ALP, PPAR-γ, leptin and annexin V) was normalised against β-2-microglobulin and calculated using the 2^-ΔΔCt^ method. Sequences of all primers are shown in Table 1.

**Table 1:** Primer Sequences for Qualitative Real-Time Polymerase Chain

Gene	Primer nucleotide sequence
Shh	Forward: CTGACCGTGACCGTAGCAAGT Reverse: TGGATGTGGGCTTTGGACTCA
Sox 9	Forward: CTGACCGTGACCGTAGCAAGT Reverse: TGGATGTGGGCTTTGGACTCA
ANCN	Forward: ATGGCTTCCACCAGTGCG Reverse: CGGATGCCGTAGGTTCTCA
collagen II	Forward: GCTCCCAGAACATCACCTACCA Reverse: ATTCCTGCTCAGGCCCTCC
collagen X	Forward: CCCTTCTGCTGCTAGTGTC Reverse: GTCTTGGTGTTGGGTTGT
RUNX2	Forward: CCTTCCACTCTCAGTAAGAAGA Reverse: TAAGTAAAGGTGGCTGGATAGT
ALP	Forward: CCTCTTGGGTCTCTTTGAGC Reverse: CAATCCTGCCTCCTTCCA
PPAR-γ	Forward: GCATCCCCACCCTACTATTCTG Reverse: GAGGGAGTTGGAAGGCTCTTC
leptin	Forward: GTCGTCGGTTTGGACTTCATC Reverse: CGGAGGTTCTCCAGGTCGTTG
Annexin V	Forward: GCAGAACTAACAGCCATAA Reverse: AGAACCACCAACATCCTC
B2M	Forward: AACGTGGAACAGTCAGACC Reverse: AGTAATCTCGATCCCATTTC

### Western blotting

Expression of hedgehog signalling molecules and cartilage-related proteins was determined by western blotting as described previously [14, 23]. Antibodies recognising Ptc (Aviva Systems Biology, San Diego, CA, USA; 1:500), Smo (Aviva Systems Biology; 1:1,600), Gli1 (Biorbyt, Cambridgeshire, UK; 1:1,000), Sox9 (OriGene, Rockville, MD, USA; 1:500), collagen II (Novus Biologicals, Littleton, CO, USA; 1:200), ACAN (Novus Biologicals; 1:100), collagen X (Abcam; 1:500), RUNX2 (Abcam; 1:500), ALP (Abcam; 1:500), PPAR-γ (Santa Cruz Biotechnology; 1:1000) and GAPDH (Novus Biologicals; 1:2000) were used as primary antibodies. The secondary antibody (1:2000) was purchased from Thermo (Waltham, MA, USA). GAPDH was used as an internal control protein.

### Apoptosis during differentiation

Annexin V, a marker of apoptosis and hypertrophy, was evaluated as described in a previous study [18]. Apoptotic and hypertrophic cells were stained using an annexin V-PE detection kit (Beyotime, Shanghai, China) based on the manufacturer’s protocol after 21 days of induction. Briefly, single labelling was performed using the red fluorochrome phycoerythrin (PE) that binds to apoptotic and hypertrophic cells. Apoptotic cells were evaluated in representative sections using a fluorescence microscope.

### Surgical technique

Twenty-four adult female New Zealand white rabbits weighing 2.5–3 kg was used according to protocols approved by the Care of Experimental Animals Committee of Tenth People’s Hospital of Tongji University (SHDSYY-20170623). Rabbits were housed with an inverse 12 h day-night cycle with lights in a temperature (22 ± 1 °C) and humidity (60 ± 5%) controlled room. All rabbits are raised in separate cages. The following surgical procedure was performed as previously described [29]. All operations were performed under sterile conditions. Anaesthesia was maintained by intramuscular injection of pentobarbital (30 mg/kg), and a full-thickness osteochondral defect (diameter: 4 mm, depth: 5 mm) was created with a surgical drill bit in the centre of the trochlear groove of the left leg. A line marked on the drill bit was used to limit the depth. After 3 days of expansion in RCCS, undifferentiated BMSCs were digested from microcarriers. Undifferentiated BMSCs (1 × 10^6^) were implanted at the defect site using fibrin glue (Tisseel, Baxter AG, Vienna, Austria). Rabbits were randomized into three groups according to the implant: group 1, fibrin glue containing Shh-transfected BMSCs; group 2, fibrin glue containing non-transfected BMSCs; group 3, fibrin group. Rabbits were able to move freely in the cage after surgery, and their basic health status was supervised by a veterinarian.

At 6 and 12 weeks after surgery, four rabbits from each group were euthanised by injection of thiopentone. After sacrifice, the femoral condyles were collected for histological assessment and immunohistochemical analysis. Before sacrifice, all rabbits were healthy and showed no signs of knee infection.

### Histological assessment and immunohistochemical analysis

Cells cultured in the RCCS three-dimensional environment were fixed in 4% (w/v) paraformaldehyde after washed with PBS for 30 min. Cells were stained with Toluidine Blue staining solution according to standard protocols. Engineered cartilage samples from each animal were fixed in 10% neutral-buffered formalin, decalcified, and embedded in paraffin. Sections (5 μm thick) were used for staining with HE for routine histological evaluation. Toluidine Blue staining was used to evaluate the content of proteoglycans in the cartilage matrix. Histopathological assessment was performed according to the Visual Histological Assessment Scale [30].

In vitro and in vivo samples were harvested for immunohistochemical analysis of collagen II (Novus) according to the method escribed previously [31]. The dilution of primary antibody recognising collagen II was 1:100.

### Statistical methods

All data are expressed as the mean ± SD with repeated at least three times and yielded similar results. The significance of differences among groups was tested by analysis of variance (ANOVA) or non-parametric tests using SPSS 19 statistical software (SPSS, Inc., Chicago, IL, USA). Multiple comparisons between groups were performed by ANOVA followed by Student Newman-Keuls multiple comparison tests. A *p*-value < 0.05 was considered significant.

## Data Availability

The datasets used and analysed during the current study are available from the corresponding author on reasonable request.

## Abbreviations

3D: Three-dimensional
ACAN: Aggrecan
ANOVA: Analysis of variance
B2M: β-2-Microglobulin
BMSCs: Bone marrow stromal cells
ECM: Extracellular matrix
ELISA: Enzyme-linked immunosorbent assay
GFP: Green fluorescence protein
HE: Hematoxylin and eosin
ITS: Insulin-transferrin-selenium solution
OA: Osteoarthritis
pfu: plaque-forming units
Ptc: Patched
qRT-PCR: quantitative real-time PCR PBS Phosphate-buffered saline
RCCS: Rotary cell culture system
Shh: Sonic hedgehog
Smo: Smoothened
